# Uukuniemi Phlebovirus Assembly and Secretion Leave a Functional Imprint on the Virion Glycome

**DOI:** 10.1128/JVI.01662-14

**Published:** 2014-09

**Authors:** Max Crispin, David J. Harvey, David Bitto, Steinar Halldorsson, Camille Bonomelli, Matthew Edgeworth, James H. Scrivens, Juha T. Huiskonen, Thomas A. Bowden

**Affiliations:** a Oxford Glycobiology Institute, Department of Biochemistry, University of Oxford, Oxford, United Kingdom; b Division of Structural Biology, Wellcome Trust Centre for Human Genetics, University of Oxford, Oxford, United Kingdom; c School of Life Sciences, University of Warwick, Coventry, United Kingdom

## Abstract

Uukuniemi virus (UUKV) is a model system for investigating the genus Phlebovirus of the Bunyaviridae. We report the UUKV glycome, revealing differential processing of the Gn and Gc virion glycoproteins. Both glycoproteins display poly-*N*-acetyllactosamines, consistent with virion assembly in the medial Golgi apparatus, whereas oligomannose-type glycans required for DC-SIGN-dependent cellular attachment are predominant on Gc. Local virion structure and the route of viral egress from the cell leave a functional imprint on the phleboviral glycome.

## TEXT

The genus Phlebovirus, in the family Bunyaviridae, includes approximately 70 arboviruses with a near-worldwide distribution, many of which are zoonotic and of significance to human health (1). Although Rift Valley fever virus (RVFV) is endemic in many parts of Africa and the Middle East (2, 3), other phleboviruses, such as the Heartland virus (HRTV) (4–6) and severe fever with thrombocytopenia syndrome virus (SFTSV) (7, 8), have only recently emerged in North America and China, respectively. The emergence of the highly pathogenic SFTSV (fatality rates near 30%), for example, has been rapid, with known lineages originating approximately 100 to 150 years ago (9). Although this genus is a subject of intensive research (10–15), there are currently no specific therapeutics to prevent or treat Phlebovirus infection in humans. Furthermore, a detailed understanding of the posttranslational modifications of the virion surface is lacking.

Uukuniemi virus (UUKV) was first isolated in 1960 from ticks in southeast Finland (16) and has been adopted as a prototype for studying phlebovirus ultrastructure (17, 18) and pathobiology (19). In addition to ticks, UUKV has been serologically detected in humans, cows, birds, reindeer, and rodents (20, 21). Like all other phleboviruses, UUKV is enveloped and possesses a negative-sense genome which is divided into three segments, S, M, and L. The glycoprotein precursor, which is encoded by the M segment, is cotranslationally cleaved in the endoplasmic reticulum (ER) by cellular proteases into two transmembrane glycoproteins, Gn (∼70 kDa) and Gc (∼65 kDa), both of which are required for host cell entry (22). X-ray crystallographic analysis of the Gc glycoprotein from the closely related RVFV has revealed a class II fusion glycoprotein architecture (23). The structure of the Gn glycoprotein remains unknown.

Phleboviruses enter host cells through receptor-mediated endocytosis (24, 25). Entry into mammalian dendritic cells is thought to be instigated through an initial viral attachment interaction between virion glycoprotein-associated oligomannose-type glycans and a tetrameric C-type lectin, DC-SIGN (26, 27). UUKV Gn and Gc both contain four N-linked glycosylation sequons. It is unknown whether both viral glycoproteins are involved in lectin-mediated cellular attachment.

Studies using baby hamster kidney 21 (BHK-21) cells as a model system to examine posttranslational modifications on UUKV have revealed that both Gn and Gc contain endoglycosidase H-resistant and -sensitive N-linked glycans (26, 28, 29). Following upon these earlier electrophoretic analyses, we performed a complete glycomic analysis of the N-linked glycans displayed by UUKV. UUKV was propagated by infection of BHK-21 cells at a multiplicity of infection of 0.1, and the cells were maintained with Glasgow minimal essential medium supplemented with 10% tryptose phosphate broth and 5% fetal bovine serum at 37°C in an atmosphere containing 5% CO_2_. Media containing secreted UUKV were collected 42 h following infection. Cell supernatants were clarified and virions were concentrated by ultracentrifugation through a 20% sucrose cushion, as previously described (30). Virus pellets were resuspended in neutral-pH buffer.

**FIG 1:**
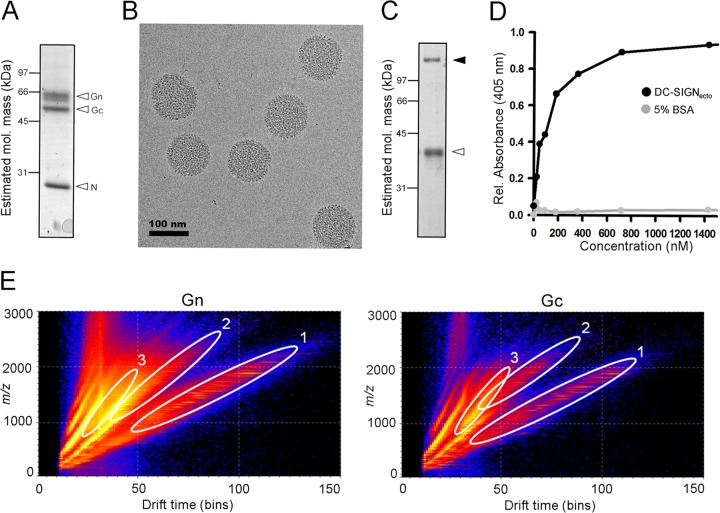
UUKV preparation, DC-SIGN binding and glycan isolation. (A) SDS-PAGE analysis with Coomassie staining revealing protein bands corresponding to the structural proteins Gn, Gc, and N. (B) Cryo-EM of purified UUKV virions. Purified UUKV from BHK-21 cells was vitrified by rapid plunge-freezing on electron microscopy grids (C-flat; Protochips, Raleigh, NC, USA) into liquid ethane. Electron cryomicroscopy was performed using a 300-kV transmission electron microscope (F30 Polara; FEI, Eindhoven, Netherlands) operated at a temperature of approximately 100 K. Images of UUKV were taken at −5 μm defocus using a charge-coupled device (CCD) camera (Ultrascan 4000SP; Gatan, Pleasanton, CA) at a nominal magnification of 59,000×, corresponding to a calibrated pixel size of 0.2 nm with a dose of approximately 20 e^−^/Å^2^. (C) Reducing SDS-PAGE analysis with Coomassie staining of purified DC-SIGN ectodomain (DC-SIGN_ecto_), revealing two bands corresponding to monomeric (white arrowhead) and putative residual, tetrameric (black arrowhead) species. DC-SIGN_ecto_ containing the C-terminal carbohydrate recognition motif and five tandem repeats (residues 135 to 404; UniProt accession number Q9NNX6, synthesized by GeneArt) was cloned into the pHLsec vector and expressed in human embryonic kidney (HEK) 293S cells (58, 59). His-tagged DC-SIGN_ecto_ was purified by metal affinity and size exclusion chromatography. (D) ELISA plot showing the interaction between tetrameric DC-SIGN_ecto_ and immobilized UUKV virions. Binding of UUKV to DC-SIGN_ecto_ was determined by ELISA in the presence of 20 mM HEPES buffer containing 100 mM NaCl, 2 mM CaCl_2_. UUKV in complex with DC-SIGN_ecto_ was detected with a rabbit anti-hexahistidine antibody (ab9108; Abcam, Cambridge, United Kingdom). Horseradish peroxidase-conjugated goat anti-rabbit IgG (PI-1000; Vector Laboratories, Peterborough, United Kingdom) was added to the sandwich ELISA for detection using the ABTS [2,2′-azinobis(3-ethylbenzthiazolinesulfonic acid)] kit (Vector Laboratories). (E) Driftscope plot (drift time against *m/z*, negative ions, log scale) of N-linked glycans from the UUKV Gn (left) and Gc (right) glycoproteins. Regions occupied by singly (oval 1), doubly (oval 2), and triply (oval 3) charged glycan ions contain the ions displayed and analyzed in subsequent figures.

The purity and sample integrity of concentrated UUKV virions were verified by electrophoretic analysis (Fig. 1A) and electron cryo-microscopy (cryo-EM) (Fig. 1B), respectively. Consistent with previous structural analyses of phleboviruses (17, 31–33), electron micrographs revealed spherical virions, with glycoprotein spikes extending from the viral membrane. Binding of purified UUKV virions to recombinant DC-SIGN ectodomain was confirmed by ELISA, demonstrating the functional integrity of the virions in the context of receptor recognition (Fig. 1C and D). These data confirmed that our UUKV was of sufficient quality and purity to warrant mass-spectrometric analysis of virion-associated N-linked glycosylation (Fig. 1E).

**FIG 2:**
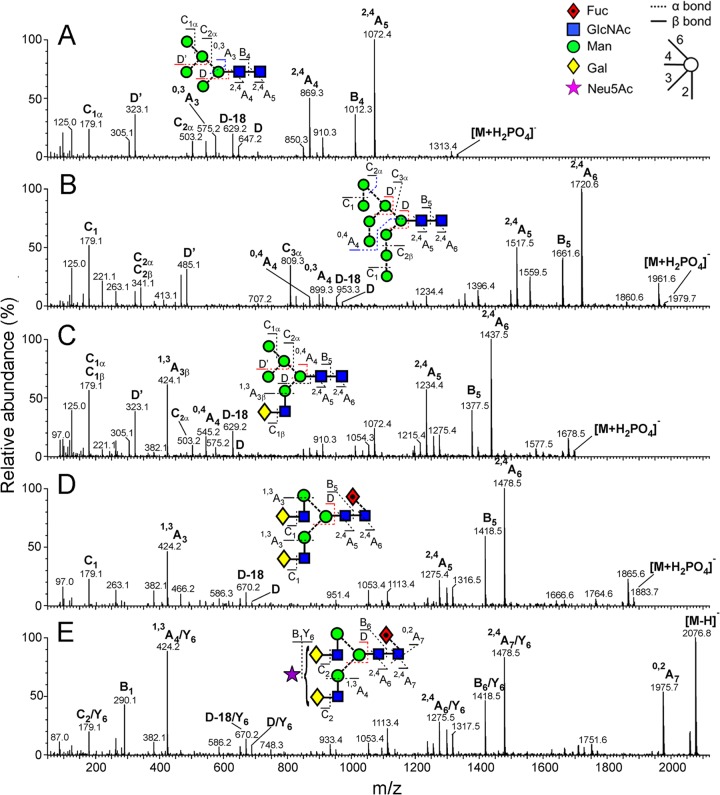
Examples of mobility-extracted, negative ion CID (transfer region) spectra of representative N-linked glycans from UUKV. (A) Man_5_GlcNAc_2_; (B) Man_9_GlcNAc_2_; (C) Man_5_GlcNAc_3_Gal_1_; (D) Man_3_GlcNAc_4_Gal_2_Fuc_1_; (E) Man_3_GlcNAc_4_Gal_2_Fuc_1_Neu5Ac_1_. A key to the symbols used for the glycan structures is displayed in the upper right hand corner of panel A (60). Ion nomenclature follows that proposed by Domon and Costello (61) with spectral interpretation performed as described by Harvey et al. (38, 40).

To study the N-linked glycome of UUKV, SDS-PAGE gel bands corresponding to the Gn and Gc glycoproteins were excised and digested with PNGase F, as previously described (34, 35). Gn and Gc glycans were subjected to ion mobility mass spectrometry (electrospray ionization) and collision-induced dissociation (CID) analysis, a highly sensitive method which can separate contaminating compounds resulting from the extraction process (36). Mass spectrometry was carried out in negative-ion mode with a Waters Synapt G2 traveling wave ion mobility mass spectrometer (Waters MS Technologies, Manchester, United Kingdom). We resolved singly, doubly, and triply charged glycan ions by the computational mining of a Waters Driftscope plot, which displays *m/z* versus drift time (Fig. 1E). The mass of each glycan was used as a fingerprint to reveal monosaccharide composition. Negative-ion fragmentation analysis confirmed these assignments and enabled accurate assignment of isomers (37–40). A representative analysis of five glycan ions is presented in Fig. 2.

**FIG 3:**
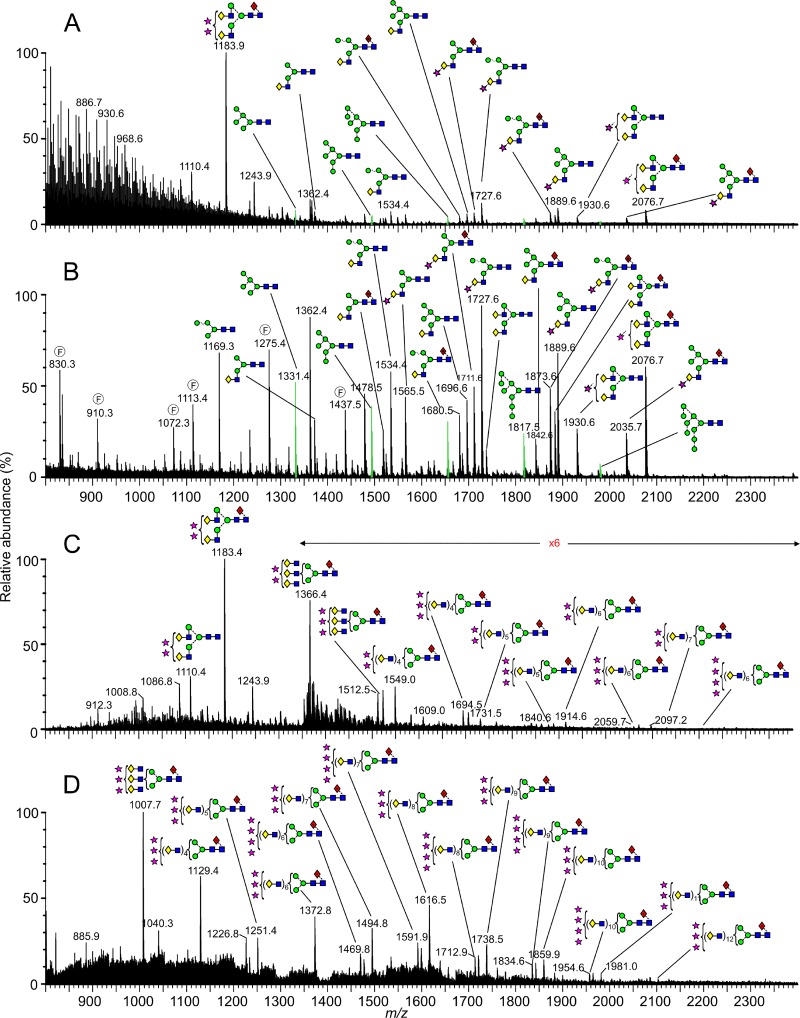
Mass-spectrometric analysis of N-linked glycans from UUKV Gn. (A) Raw electrospray ionization spectrum; (B to D) corresponding spectra of isolated glycans with singly (B), doubly (C), and triply (D) charged ions. Fragment ions are annotated with an encircled F. Neutral glycans form [M+H_2_PO_4_]^−^ ions. Sialylated glycans form [M−H]^−^ (singly charged), [M−H_2_]^2−^ (doubly charged), and [M−H_3_]^3−^ (triply charged) ions. Peaks corresponding to Man_5–9_GlcNAc_2_ are green. Symbols used for glycan structures are defined in the legend to Fig. 2.

**FIG 4:**
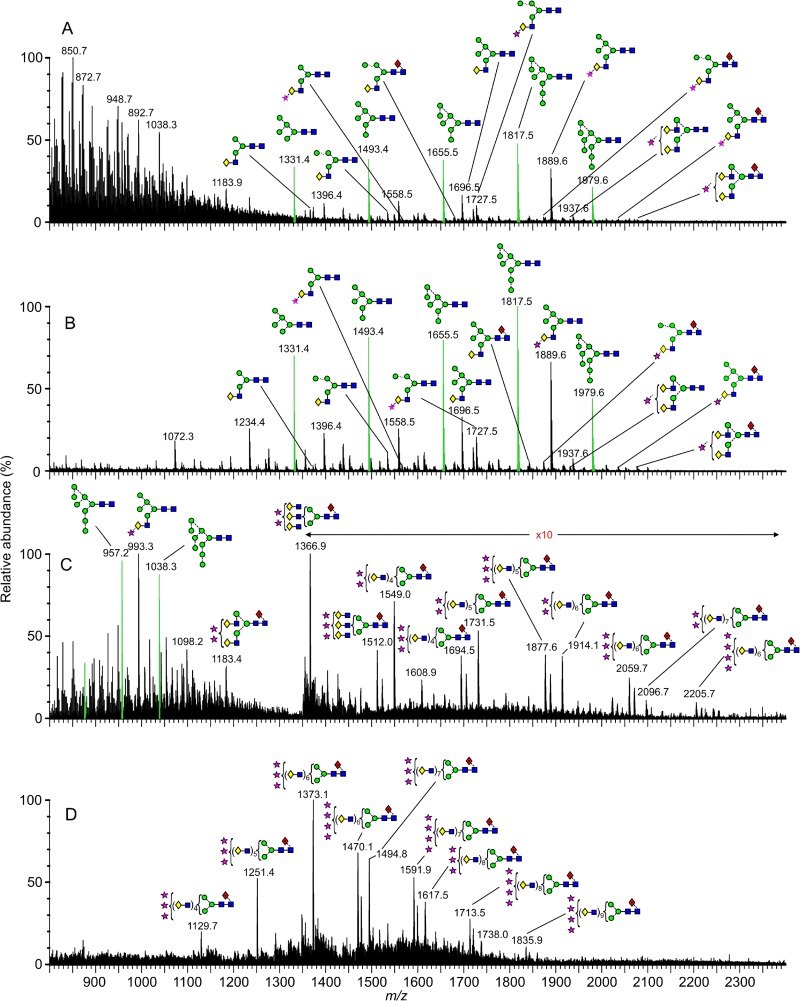
Mass-spectrometric analysis of N-linked glycans from UUKV Gc. (A) Raw electrospray ionization spectrum; (B to D) corresponding spectra of isolated glycans with singly (B), doubly (C), and triply (D) charged ions. Neutral glycans form [M+H_2_PO_4_]^−^ ions. Sialylated glycans form [M−H]^−^ (singly charged), [M−H_2_]^2−^ (doubly charged), and [M−H_3_]^3−^ (triply charged) ions. Peaks corresponding to Man_5–9_GlcNAc_2_ are green. Symbols used for glycan structures are defined in the legend to Fig. 2.

The ESI mass spectra of the isolated Gn and Gc glycans are shown in Fig. 3 and 4, respectively, and reveal that although both glycoproteins display a mixture of fully processed and underprocessed glycans, Gc has a greater level of underprocessed glycans than Gn (Man_5–9_GlcNAc_2_; green peaks in Fig. 3 and 4). The total spectrum of Gn was dominated by complex-type structures (Fig. 3A), with a prominent population of disialylated, core-fucosylated biantennary glycans (*m/z* 1,183.9). Smaller populations of monosialylated mono- and biantennary structures were also observed, together with a number of hybrid- and oligomannose-type structures. This spectrum contrasts that of the total spectrum of Gc (Fig. 4A), which had only a minor population of disialylated complex-type glycans and larger populations of lesser-processed hybrid- and oligomannose-type glycans (e.g., Man_5_Gal_1_GlcNAc_3_ and Man_5–9_GlcNAc_2_, respectively). These differences in glycan composition are also reflected in our electrophoretic analysis, where the relatively sharp band of Gc correspondingly exhibited more homogenous and less biosynthetically processed glycans than the more diffuse band of Gn (Fig. 1A).

Mining of the computational Driftscope plot (Fig. 1E) demonstrated that many glycan species were suppressed in the total ion spectra (Fig. 3A and 4A), particularly in the analysis of Gn. Analysis of singly charged populations (Fig. 3B and Fig. 4B) enabled detailed examination of glycans that were obscured by the more dominant populations. The improved signal-to-noise ratio achieved by this extraction process enabled additional low-abundance structures to be detected, and we were able to resolve a minor population of oligomannose-type structures on Gn (e.g., *m/z* 1,817.5). These results reveal that Gc displays a greater abundance of oligomannose-type glycans, yet both Gn and Gc exhibit ligands for DC-SIGN. However, it is unknown which of these are accessible for lectin recognition during cell entry.

Steric occlusion of N-linked glycans can impose compositional constraints upon the viral glycome through the inhibition of glycan biosynthesis in the host cell (35). This can be achieved through intramolecular glycan-glycan or glycan-protein interactions, as illustrated by the glycosylation of gp120 from human immunodeficiency virus type 1 (HIV-1) (41). Similarly, glycan-protein interactions have a functional role for dengue virus, leading to the formation of oligomannose-type glycans and a productive interaction with DC-SIGN (42).

It is possible to postulate which enzymatic steps are most sensitive to disruption due to the physical presentation of glycans during UUKV biogenesis. In contrast to the competing reactions that occur in the medial and late Golgi apparatus, the early stages of glycan processing in the ER and cis-Golgi are comparably linear (43). In our analysis of both UUKV Gn and Gc, there is no evidence that any one step of glycan biosynthesis is absolutely inhibited. However, the presence of the Man_6–9_GlcNAc_2_ series, particularly on Gc, indicates reduced sensitivity to ER α-mannosidase I and Golgi α-mannosidases IA to -C (Fig. 3 and 4). Similarly, the presence of Man_5_GlcNAc_2_ indicates an inefficiency of GlcNAc transferase (GnT) I (Fig. 3 and 4). Finally, the presence of hybrid-type glycans on Gc, which are typically at very low abundance, can be attributed to lessened sensitivity to Golgi α-mannosidase II (43). Our comparison of Gn and Gc glycan composition shows that Gn exhibits more highly processed glycans than Gc and is therefore more likely to be accessible to processing enzymes.

Due to the presence of complex-type glycans on both glycoproteins, we deduce that the underprocessed glycans arise as a result of steric occlusion, rather than differential transit through the host cell. Driftscope analysis of doubly and triply charged ions enabled identification of glycan structures not previously reported for phleboviruses (Fig. 3C and D; Fig. 4C and D). In both spectra of Gn and Gc glycans, we observed a small population of large and highly processed poly-*N*-acetyllactosamine extensions. Poly-*N*-acetyllactosamines have been observed on the nonstructural NS1-B glycoprotein of influenza B virus, on a variety of isolates derived from a range of cell types (44). Such structures have also been observed on macrophage-derived HIV-1 (45) and HIV-2 (46) as well as on the small hydrophobic protein of human and bovine respiratory syncytial viruses derived from bovine nasal turbinate cells and HEp-2 cells, respectively (47).

Poly-*N*-acetyllactosamine structures arise through the dual action of medial-Golgi apparatus-resident β1-4-galactosyltransferase and GnT V (which catalyzes the transfer of GlcNAcβ1-6 to galactose). Although no functional role has been ascribed to phleboviral poly-*N*-acetyllactosamine structures, we suggest that their presence provides a marker for phlebovirus biosynthesis and is indicative of prolonged glycan residence time in the medial Golgi apparatus (48). This hypothesis is supported by previous studies by Nabi et al., which revealed a direct correlation between the rate of Golgi-residence egress and the extent of poly-*N*-acetyllactosamine formation (49, 50). This observation is entirely consistent with established pathways of UUKV glycoprotein biosynthesis and assembly, where Gn-Gc heterodimers (51), produced in the ER, are retained in the medial Golgi apparatus as a result of a Golgi retention signal in the cytoplasmic tail of Gn (52, 53). Such prolonged localization during phleboviral assembly is also consistent with viral budding taking place in the ERGIC (ER-Golgi intermediate compartment) and Golgi apparatus (54, 55).

Here, through the application of ion mobility mass spectrometry, we have structurally characterized the glycans presented on the Gn and Gc glycoproteins from the mature UUKV virion. Surprisingly, we observed that both glycoproteins display a range of glycans spanning from ER-associated oligomannose-type to large and highly processed poly-*N*-acetyllactosamine structures. We show that even though the major class of glycosylation on Gn was of the highly processed complex type, there were small populations of hybrid- and oligomannose-type glycans. In contrast, Gc was dominated by DC-SIGN-binding, oligomannose-type glycans, with remaining structures primarily of the hybrid type.

Viral glycan structure and composition are key determinants in virus-host pathobiology and may be modulated by both the virus and the host (35). In the case of UUKV phlebovirus, our analysis defined populations of mannose-terminating glycan structures on the virion surface and demonstrated direct recognition of the virion by DC-SIGN, consistent with C-type lectin cell attachment (26). We also observed two examples of differential processing of the Gn and Gc glycoproteins. First, despite exiting more slowly from the ER than Gn (28) and thus having longer exposure to α-mannosidases, Gc showed a higher proportion of oligomannose-type glycans than Gn. Second, despite being ostensibly exposed to processing enzymes during virion assembly in the Golgi apparatus, Gc exhibited somewhat smaller poly-*N*-acetyllactosamine extensions than Gn. These observations lead us to propose that structural constraints limit UUKV glycan processing, predominantly on the Gc glycoprotein.

There is an interesting paradox that steric occlusion drives the formation of oligomannose-type glycans while not precluding the productive interaction of these same glycans with cellular receptors. This may arise because glycan processing is highly sensitive to intramolecular glycan-protein interactions. For example, the occlusion of a single glycan branch may prevent processing of the entire glycan, leaving functionally important termini exposed for receptor recognition. Alternatively, this phenomenon may arise because cellular receptors are smaller than glycan processing enzymes. The immunological properties of oligomannose-type glycans are also of interest. They can be potent activators of the innate immune system and as immunologically “self” structures do not elicit a strong antibody response (56). Although potent neutralizing antibodies have now been identified that recognize mixed glycan-protein epitopes (57), one may expect a limited antibody response against oligomannose-type glycans.

In conclusion, we suggest that the extremes of glycan processing result from a combination of local viral protein architecture and the route of virus egress through the cell. Given the correlation between the biosynthetic pathway and the observed glycan structures, we postulate that low-abundance poly-*N*-acetyllactosamines are a conserved feature of the Phlebovirus genus. Our glycomic analysis reveals virion-directed glycosylation strategies for host cell entry, provides a sensitive reporter for the route of virion assembly, and refines the antigenic surface of phleboviruses.
